# Nanomedicine against antimicrobial resistance: mechanistic insights and next-generation therapeutic potential

**DOI:** 10.3389/fchem.2026.1915145

**Published:** 2026-09-14

**Authors:** Vishal L. Handa, Benedict B. Vyas, Abhishek Padhi, Ashwini Agarwal, B. R. M. Vyas

**Affiliations:** 1 NOHP-PCZ, National Center for Disease Control, All India Institute of Medical Sciences, Rajkot, Gujarat, India; 2 Department of Biosciences, Saurashtra University, Rajkot, Gujarat, India; 3 Department of Clinical Microbiology and Infectious Disease, All India Institute of Medical Sciences, Rajkot, Gujarat, India

**Keywords:** antibiotic resistance, antimicrobial nanomedicine, biofilm formation, clinical pathogens, extensively drug resistant, multidrug resistant

## Abstract

Antimicrobial resistance (AMR) has emerged as one of the most critical global health threats, severely limiting the effectiveness of existing therapies against bacterial, fungal, and viral infections. The increasing prevalence of multidrug-resistant pathogens is largely driven by rapid genetic evolution, which promotes multiple resistance mechanisms such as drug degradation, target-site alteration, reduced intracellular drug accumulation, and biofilm formation, leading to persistent infections and rising mortality. These challenges highlight the urgent need for alternative therapeutic strategies beyond conventional antimicrobials. Nanomedicine has gained considerable attention due to its unique physicochemical properties, enabling improved drug stability, targeted delivery, controlled release, enhanced pathogen penetration, and multimodal antimicrobial action. This review comprehensively examines resistance mechanisms across major microbial pathogens and discusses the evolution of nanomedicine as an advanced platform for combating AMR. Particular emphasis is placed on green biogenic synthesis of nanoparticles using biological resources, offering environmentally sustainable and biocompatible antimicrobial nanomaterials. The synergistic interactions between nanomaterials and conventional antimicrobial agents that enhance therapeutic efficacy and restore susceptibility in resistant pathogens. Emerging next-generation antimicrobial nanomedicine platforms, including biomimetic nanoparticles, antimicrobial peptide delivery systems, CRISPR-enabled nanocarriers, and stimuli-responsive systems, are also highlighted for their potential in precision infection management. Finally, key challenges involving toxicity, biosafety, microbiome disruption, environmental impact, manufacturing scalability, regulatory and clinical translation. Addressing these barriers will be essential for advancing safe and effective nanomedicine-based solutions against AMR.

## 1 Introduction

Antimicrobials are natural, semi-synthetic, or synthetic agents (antibiotics, antifungals, antivirals, antiparasitics, antiseptics, *etc*.) used to treat infectious diseases (Nankervis et al., 2016). However, the rapid emergence and global spread of antimicrobial-resistant (AMR) pathogens have created a major therapeutic crisis. The cost of treating antibiotic-resistant infections ranges from $3,000 to $41,000 USD per patient in low-income and high-income nations, respectively (Naylor et al., 2025). Globally, AMR-related deaths are projected to reach 8.22 million annually by 2050 if effective interventions are not implemented; majority AMR-associated deaths are driven by major bacterial pathogens: methicillin-resistant *Staphylococcus aureus* (MRSA) predominates in high-income regions, whereas carbapenem-resistant Gram-negative pathogens, particularly *K. pneumoniae* and *Acinetobacter baumannii*, dominate mortality in South Asia, sub-Saharan Africa, and Latin America (Naghavi et al., 2024). Carbapenem-resistant *Klebsiella pneumoniae*, *A*. *baumannii*, *Pseudomonas aeruginosa*, rifampicin-resistant *Mycobacterium tuberculosis*, MRSA, and vancomycin-resistant *Enterococcus faecium*, *Escherichia coli* are ranked among the highest priorities due to their significant clinical burden, increasing resistance, limited treatment options, and substantial public health impact (Handa et al., 2024; WHO BPPL, 2024).

Beyond bacterial pathogens, antifungal resistance has also emerged as a major global therapeutic concern, particularly among immunocompromised individuals and hospitalized patients. Globally, 6.5 million serious fungal infections occur annually including chronic aspergillosis (1.5 million), invasive aspergillosis (>2 million), candidemia and invasive candidiasis (1.6 million), and pneumocystis pneumonia (>0.4 million), representing the highest disease burden. Despite treatment, fungal infections contribute to approximately 3.75 million deaths annually, of which nearly 2.55 million are directly attributable to fungal infections, highlighting their substantial yet underrecognized global health burden (Denning, 2024). *Cryptococcus neoformans*, *Candida auris*, *Aspergillus fumigatus*, and *Candida albicans* are critical-priority fungal pathogens due to their high mortality, increasing incidence, limited therapeutic options, and growing antifungal resistance (WHO FPPL, 2022). Consequently, invasive fungal infections, particularly in immunocompromised individuals, have become an increasing therapeutic challenge.

Similarly, antiviral resistance in clinically important and emerging viral pathogens increasingly limits treatment effectiveness. Viral pathogens include coronaviruses (MERS-CoV, SARS-CoV-2), influenza A viruses (H1, H3, H5, H7, H9, *etc*.), filoviruses (*Orthoebolavirus zairense* and *Orthomarburgvirus marburgense*), flaviviruses (dengue and Zika viruses), henipaviruses (Nipah virus), hantaviruses, orthopoxviruses (mpox virus), hemorrhagic fever viruses (Crimean-Congo hemorrhagic fever virus) and Rift Valley fever virus, many of which present growing therapeutic concerns due to limited antiviral options and emerging resistance risks (WHO, 2024). Notably, SARS-CoV-2 has emerged against remdesivir within a year of FDA approval, while resistance risks to molnupiravir and 3CL protease inhibitors remain a growing therapeutic concern (Dinata et al., 2025). Collectively, the increasing prevalence of multidrug-resistant (MDR), extensively drug-resistant (XDR), and pan-drug-resistant pathogens across bacterial, fungal, and viral species has exposed major limitations of conventional antimicrobial therapies. Despite continuous antimicrobial discovery and development efforts, the therapeutic pipeline remains insufficient to meet the escalating global burden of resistant infections. Therefore, there is an urgent need for innovative therapeutic platforms capable of improving antimicrobial delivery, overcoming resistance mechanisms, and restoring the efficacy of existing antimicrobial agents.

Nanomaterials, including metallic, polymeric, lipid-based, and green-synthesized nanoparticles, have demonstrated broad-spectrum antimicrobial activity either alone or in combination with conventional antimicrobial agents, thereby improving therapeutic efficacy while reducing the risk of resistance development (Gangadhar and Subburaj, 2025). Nanomedicine has emerged as a promising next-generation therapeutic strategy due to its unique physicochemical properties, targeted drug delivery capabilities, enhanced antimicrobial penetration, and biofilm disruption potential (Thakur et al., 2025). Several recent reviews have examined the use of nanomedicine to address AMR, including metallic and polymeric nanomaterials, biogenic nanoparticles, and nanotechnology-enabled CRISPR-Cas delivery systems (Abdallah et al., 2026; Ahmed M. E. et al., 2025). However, these reviews have largely focused on specific nanomaterial classes or therapeutic platforms. An integrated framework linking resistance mechanisms across bacterial, fungal, and viral pathogens with nanomedicine design, adaptive resistance, synergistic applications, and clinical translation remains limited.

Accordingly, this review examines how nanomedicine can address diverse AMR mechanisms through enhanced delivery, multimodal activity, biofilm penetration, and synergistic combinations. We further discuss the evolution of antimicrobial nanomedicine from conventional nanocarriers to biogenic, biomimetic, stimuli-responsive, and CRISPR-enabled platforms, while critically evaluating adaptive resistance, toxicity, biosafety, manufacturing, regulatory, and translational challenges. This integrated perspective highlights key opportunities and limitations for developing sustainable and clinically relevant nanomedicine-based solutions to AMR.

## 2 Mechanistic insights into inhibition of AMR pathogens

Microorganisms including bacteria, fungi, and viruses employ diverse molecular and physiological mechanisms including natural (inherent), adaptive, and acquired resistance mechanisms (Belay et al., 2024).

### 2.1 Antimicrobial resistance mechanisms in bacteria

In bacteria, antimicrobial resistance primarily occurs through several key mechanisms, including enzymatic degradation and/or modification of antibiotics, target sites alteration, and reduced intracellular drug accumulation by altered membrane permeability or porin loss, alongside increased efflux mediated antimicrobial expel (Halawa et al., 2024; Ho et al., 2025). In major Gram-negative pathogens, including *E*. *coli*, *A*. *baumannii*, *K*. *pneumoniae*, and *P. aeruginosa*, *β-*lactamases, carbapenemases, and aminoglycoside-modifying enzymes represent important mechanisms of antimicrobial agent inactivation (Kouta et al., 2026; Wang et al., 2025). In addition to enzymatic drug inactivation, overexpression of multidrug efflux systems contributes substantially to resistance by reducing intracellular antimicrobial agent concentration (Li W. R. et al., 2025; Rouvier et al., 2025; Zack et al., 2024). Mutations affecting antimicrobial targets and regulatory pathways, together with alterations in porins and other outer-membrane components, further contribute to MDR and XDR phenotypes in clinically important Gram-negative pathogens (Gauba and Rahman, 2023; Zdarska et al., 2026; Zhou et al., 2023). Although both Gram-negative and Gram-positive bacteria employ target modification, enzymatic drug inactivation, efflux systems, and biofilm formation to resist antimicrobial agents, Gram-negative bacteria are additionally protected by an outer membrane containing lipopolysaccharides, porin-mediated permeability barriers, and highly efficient multidrug efflux pumps, whereas Gram-positive bacteria predominantly rely on target-site alterations, acquisition of resistance genes such as *mecA* and *van* operons, cell wall remodeling, cell membrane modifications to reduce antimicrobial susceptibility (Assoni et al., 2020; Gauba and Rahman, 2023; Jubeh et al., 2020). Among Gram-positive pathogens, *Enterococcus* spp. and *S. aureus* are important reservoirs of antimicrobial resistance determinants. *Enterococcus* spp. exhibit resistance through altered cell-wall synthesis, aminoglycoside modifications, target-site mutations, cell-envelope stress responses, and efflux mechanisms, whereas *S. aureus* develops resistance through *β-*lactamase production, altered penicillin-binding proteins, aminoglycoside modifications, target alteration, multidrug efflux, and biofilm-associated persistence (Guo et al., 2020; Lu et al., 2025; Mancuso et al., 2021; Rajput et al., 2024). *β-*Conventional antibiotics often fail to eradicate bacterial pathogens because bacteria employ multiple defense mechanisms (Figure 1A). Therefore, controlling such pathogens requires alternative therapeutic strategies capable of simultaneously targeting multiple resistance mechanisms, which may improve treatment efficacy and reduce the likelihood of resistance development.

**FIGURE 1:**
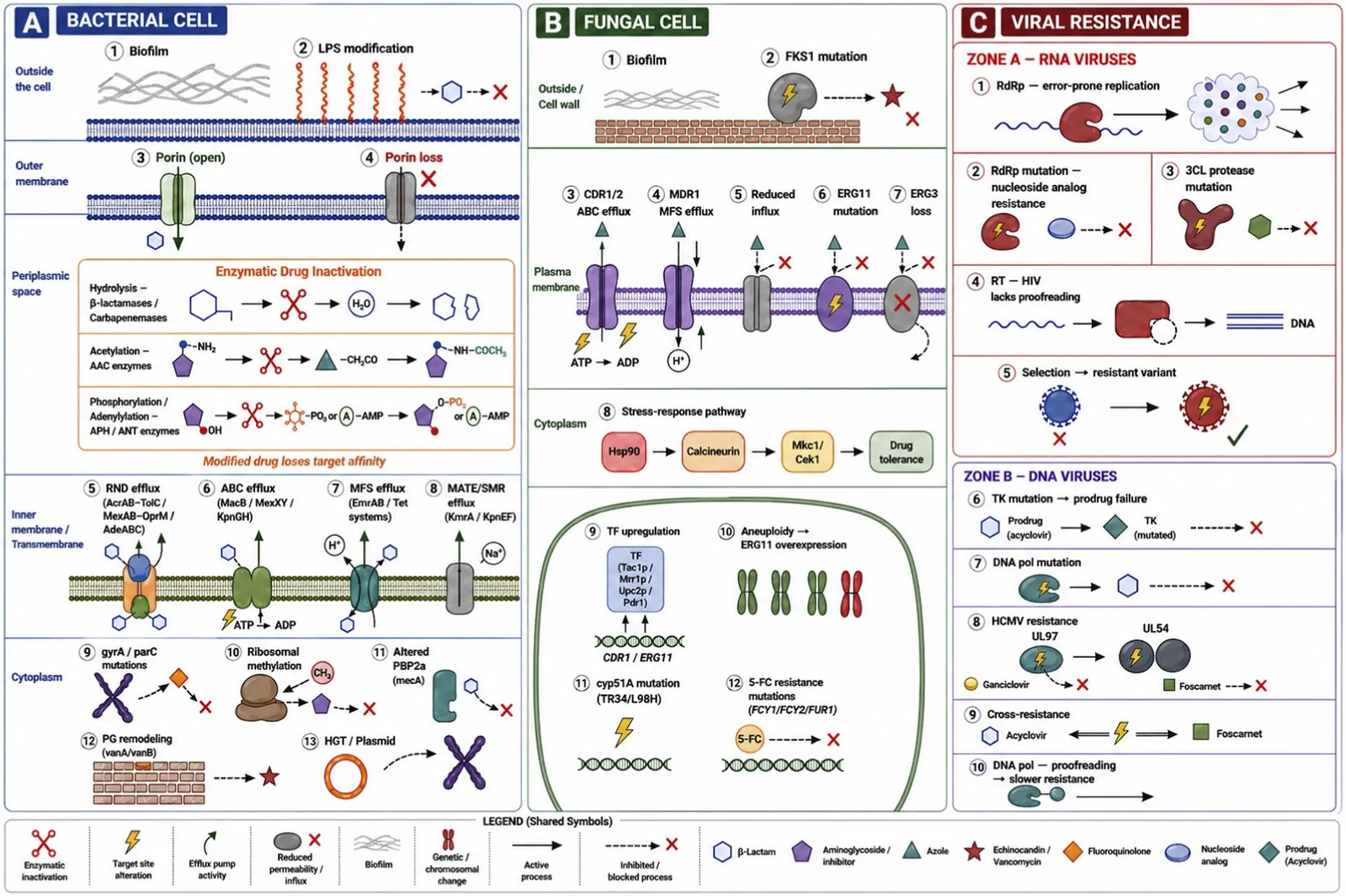
Major mechanisms of antimicrobial drug resistance in bacteria, fungi, and viruses. Schematic representation of key resistance pathways across microbial pathogens. **(A)** Bacterial resistance involves biofilm formation, reduced permeability, enzymatic drug inactivation, efflux pump activation, target modification, and horizontal gene transfer. **(B)** Fungal resistance includes biofilm-mediated protection, target gene mutations, efflux pump overexpression, reduced drug influx, stress-response signaling, and genomic adaptation. **(C)** Viral resistance arises from high mutation rates, error-prone replication, target-site mutations, and selection of drug-resistant variants. Together, these mechanisms drive antimicrobial failure and complicate treatment.

### 2.2 Drug resistance mechanisms in fungi

Drug resistance in fungi is mediated by various mechanisms including target-site alterations, drug targets overexpression, altered membrane permeability, biofilm formation, chromosomal aneuploidy, and activation of ABC transport and MFS efflux pumps, collectively reducing susceptibility to major antifungal agents and promoting multidrug resistance (Lee et al., 2023; Osset-Trénor et al., 2023). Effectiveness of azoles, amphotericin B, and echinocandins has substantially declined due to increasing resistance in *C*. *auris*, *C. albicans*, *Candida tropicalis*, *C. parapsilosis*, *Nakaseomyces glabratus*, *Trichophyton indotineae*, and *A. fumigatus*, with *C. auris* exhibiting particularly high fluconazole resistance (≥87%), reduced susceptibility to amphotericin B (8%–35%), and emerging echinocandin resistance (up to 8%), alongside increasing reports of pan-resistant isolates (WHO FPPL, 2022; van Rhijn et al., 2024).

Azole resistance in *Candida* species involves enhanced drug efflux through ABC and MFS transporters, alterations or overexpression of the azole target *ERG11*, changes in sterol biosynthesis, altered drug uptake, and activation of stress-response pathways (Bhattacharya et al., 2020; Lee et al., 2020; Xiong et al., 2025). Similarly, resistance in *Cryptococcus* spp. arises through a combination of point mutations, gene amplification, chromosomal duplication, altered drug transport, and stress-response pathways (Lee et al., 2023). In *Aspergillus* spp., azole resistance is primarily associated with alterations and overexpression of *cyp51A*, together with increased ABC transporter-mediated efflux and biofilm formation (Berger et al., 2017; Zubovskaia, 2025). Echinocandin resistance is relatively uncommon but has been linked to alterations in *fks1*, affecting *β-*1,3-glucan synthase activity and reducing susceptibility to echinocandins such as micafungin, caspofungin, and anidulafungin (De Francesco, 2023). *β-*Overall, the multifactorial nature of fungal drug resistance makes it particularly difficult to overcome, as multiple resistance pathways can operate simultaneously within a single pathogen (Figure 1B). This complexity not only reduces antifungal efficacy but also accelerates treatment failure and narrows the already limited antifungal therapeutic arsenal. This highlights the urgent need for novel antifungal agents and combination therapies targeting multiple resistance pathways.

### 2.3 Drug resistance mechanisms in viruses

Antiviral resistance has been reported in several clinically important RNA viruses, including HIV, dengue virus, Newcastle disease virus, SARS-CoV-2, and influenza virus, against agents such as maraviroc, brequinar, thapsigargin, remdesivir, nirmatrelvir, ensitrelvir, oseltamivir, baloxavir marboxil, etc. (Batool et al., 2025; Chander et al., 2022; Smyk et al., 2022). The high mutation rates in RNA viruses facilitate the rapid emergence of antiviral resistance, allowing resistant variants with direct or cross-resistance phenotypes to gain selective advantages and become dominant through sustained transmission (Aw et al., 2025). RNA viruses generally develop antiviral resistance more rapidly than DNA viruses because their error-prone RNA-dependent RNA polymerases or reverse transcriptase lack efficient proofreading activity and post-replicative repair mechanisms, resulting in high mutation rates (10^–5^ to 10^–3^ mutations/bp/cycle); coronaviruses are a notable exception, possessing a proofreading exonuclease that partially constrains mutation accumulation (Gribble et al., 2021; Yeo et al., 2020). Combined with rapid replication, large population sizes, frequent bottleneck events, genetic recombination, host-driven editing processes, strong antiviral selection pressure, antigenic drift, and in segmented viruses antigenic shift, these factors generate highly diverse viral populations that facilitate the emergence, selection, and transmission of drug-resistant variants (Domingo et al., 2021).

In contrast, DNA viruses slowly evolve antiviral resistance because of their lower mutation rates and the presence of higher-fidelity DNA polymerases with proofreading activities. Nevertheless, prolonged antiviral exposure can select resistant variants against nucleoside/nucleotide analogues, DNA polymerase inhibitors (foscarnet), and helicase-primase inhibitors through mutations in genes involved in antiviral activation or viral DNA replication (Dähne et al., 2025). In herpes simplex virus, resistance to nucleoside analogues can arise through mutations in viral thymidine kinase that impair antiviral activation, whereas mutations in viral DNA polymerase can reduce susceptibility to acyclovir and may confer cross-resistance to other antiviral agents (Ibrahim et al., 2026; Shankar et al., 2024). Similar resistance mechanisms have been reported in human cytomegalovirus, varicella-zoster virus, human herpesvirus 6, BK polyomavirus, and human adenoviruses through mutations affecting viral kinases and DNA polymerases, particularly in immunocompromised patients (Brunnemann et al., 2015; Chou, 2017; Chou et al., 2025; Hijano et al., 2026; Park et al., 2022). Antiviral resistance can emerge when suboptimal drug exposure fails to fully suppress viral replication, creating selective pressure that favors resistant variants. This process is shaped by viral factors, including pre-existing resistance mutations, polymorphisms, and subtype variation; host-related factors such as genetic variability, poor treatment adherence, and drug intolerance; and antiviral-related factors including limited potency and unfavorable pharmacokinetics (Figure 1C). Together, these factors accelerate viral adaptation, thereby reducing therapeutic durability and pose a major challenge to long-term antiviral control (Aw et al., 2025).

Overall, antimicrobial resistance across bacteria, fungi, and viruses reflects a highly adaptive and evolving survival strategy that enables pathogens to rapidly evade conventional therapeutics through diverse and often overlapping mechanisms (Table 1). A major limitation of current antimicrobial agents is their reliance on single-target, is insufficient to counter rapidly emerging resistance and highlights the need for next-generation therapeutic platforms capable of overcoming multiple biological barriers simultaneously. In this context, nanomedicine has emerged as a promising approach, offering multifunctional antimicrobial strategies through targeted delivery, enhanced intracellular penetration, controlled drug release, and synergistic mechanisms that may improve therapeutic efficacy against resistant pathogens.

**TABLE 1:** Representative antimicrobial resistance-associated genes, mutations, and mechanisms in bacterial, fungal, and viral pathogens.

Pathogen	Resistance mechanism	Representative genes/determinants	Major consequence	References
*E. coli*	β-lactamase production	*bla*TEM-1, *bla*CTX-M, *bla*CMY-2, *bla*KPC-2, *bla*NDM-5, *bla*OXA-181, *ampC*	Hydrolysis/inactivation of β-lactam antibiotics	Essalhi et al. (2024), Wang et al. (2025), Kouta et al. (2026)
	Aminoglycoside modification	*aac*, *ant*, *aph* families	Enzymatic modification and loss of aminoglycoside activity	Essalhi et al. (2024), Wang et al. (2025)
*A. baumannii*	Carbapenemase production	*bla*OXA-23, *bla*OXA-24, *bla*OXA-40, *bla*OXA-51, *bla*OXA-58	Carbapenem hydrolysis and reduced β-lactam susceptibility	Nikibakhsh et al. (2021)
	Metallo-β-lactamase production	*bla*KPC, *bla*NDM, *bla*IMP, *bla*VIM	Hydrolysis of carbapenems and other β-lactams	
*K. pneumoniae*	ESBL production	*bla*TEM, *bla*SHV, *bla*CTX-M	Hydrolysis of extended-spectrum cephalosporins and related β-lactams	Essalhi et al. (2024), Zdarska et al. (2026)
	Carbapenemase production	*bla*KPC, *bla*NDM-1, *bla*OXA	Carbapenem inactivation and treatment failure	
*P. aeruginosa*	β-lactamase/carbapenemase production	*ampC*, *bla*KPC, *bla*NDM, *bla*VIM, *bla*IMP, *bla*GES	Reduced susceptibility to β-lactams and carbapenems	Elfadadny et al. (2024)
Gram-negative bacteria	Efflux	*acrAB-tolC*, *acrEF-tolC*, *oqxAB-tolC*, *adeABC*, *mexAB-oprM*, *mexCD-oprJ*, *mexEF-oprN*, *mexXY*	Reduced intracellular antimicrobial concentration and multidrug resistance	Gauba and Rahman (2023) Zack et al. (2024) Li W. R. et al. (2025), Rouvier et al. (2025)
	Target modification/regulation	*gyrA*, *parC*, *nalB*, *nfxB*, *nfxC*, *pmrB*	Altered drug targets and reduced antimicrobial susceptibility	Gauba and Rahman (2023) Zhou et al. (2023), Zdarska et al. (2026)
	Reduced permeability	*omp*, *opr*, *carO*, *ompK36*, *phoE* families	Reduced antimicrobial uptake and intracellular drug exposure	Gauba and Rahman (2023) Zhou et al. (2023)
*Enterococcus* spp.	Vancomycin resistance	*vanA*, *vanB*, *vanC*	Peptidoglycan target remodeling and reduced vancomycin binding	Mancuso et al. (2021)
	Target/stress adaptation	*rpsL*, *gyrA*, *parC*, *liaFSR*, *walKR*	Altered target susceptibility and enhanced cell-envelope stress adaptation	Mancuso et al. (2021) Lu et al. (2025)
*S. aureus*	β-lactam resistance	*mecA*; β-lactamase determinants	PBP2a-mediated resistance and β-lactam inactivation	Guo et al. (2020), Rajput et al. (2024)
	Aminoglycoside modification	*aac(6′)-Ie-aph(2″)*	Enzymatic inactivation of aminoglycosides	Mancuso et al. (2021)
	Efflux	*norA*, *norB*, *mepA*, *smr*, *qacA/B*	Reduced intracellular antimicrobial concentration and multidrug resistance	Guo et al. (2020), Rajput et al. (2024)
*C. abicans* *C. tropicalis*	Azole efflux	*CDR1*, *CDR2*, *SNQ2*, *MDR1*, *FLU1*	Increased azole export and reduced intracellular drug accumulation	Lee et al. (2020), Xiong et al. (2025)
*C. albicans*	Azole target alteration/overexpression	*ERG11*	Reduced azole binding and increased target abundance	Lee et al. (2020)
*Candida* spp.	Sterol pathway alteration	*ERG3*	Reduced formation of toxic sterol intermediates during azole exposure	Bhattacharya et al. (2020)
	Stress-response regulation	*TAC1*, *MRR1*, *PDR1*	Enhanced efflux and adaptation to antifungal stress	Lee et al. (2020) Li Y. et al. (2025)
*Cryptococcus* spp.	Fluconazole target alteration/efflux	*ERG11*, *AFR1*, *MSH2*, *YAP1*	Reduced fluconazole susceptibility through altered sterol metabolism, transport, and stress adaptation	Boyce (2023), Deng et al. (2023)
	Amphotericin B resistance	*ERG11*, *HOB1*	Altered membrane sterol composition and reduced amphotericin B susceptibility	
	5-Fluorocytosine resistance	*FCY1*, *FCY2*, *FUR1*, *UXS1*, *URA6*, *UGD1*, *NRG1*	Impaired uptake or metabolism of 5-fluorocytosine	
	Echinocandin resistance	*FKS1*, *CDC50*	Altered cell-wall glucan synthesis and reduced echinocandin susceptibility	
*Aspergillus fumigatus*	Azole target alteration/overexpression	*cyp51A*	Reduced azole binding and/or increased Cyp51A abundance	Berger et al. (2017), Zubovskaia (2025)
	Azole resistance-associated mutations	TR34/L98H; TR46/Y121F/T289A; F46Y/M172V/N248T/D255E/E427K	Reduced azole susceptibility through altered Cyp51A function or expression	
	Efflux	*cdr1B*, *mdr1–4*, *abcD/E*, *atrI/B/C/F*	Increased azole efflux and reduced intracellular exposure	
*Aspergillus* spp.	Echinocandin target alteration	*fks1*; F675S	Altered β-1,3-glucan synthase and reduced echinocandin susceptibility	De Francesco (2023)
Herpes simplex virus	Impaired antiviral activation	Thymidine kinase; I54K, R222C, P173L, M85Stop, D229Stop	Reduced activation of nucleoside analogues	Shankar et al. (2024) Ibrahim et al. (2026)
	DNA polymerase alteration	R628C, E597K, A605V, W781V, Y941H	Reduced antiviral binding/activity and possible cross-resistance	
Human cytomegalovirus	Antiviral activation/target alteration	*UL97*, *UL54*	Reduced susceptibility to ganciclovir, maribavir, and related antivirals	Brunnemann et al. (2015) Chou et al. (2025)
Varicella-zoster virus	Antiviral activation/target alteration	*ORF36*, *ORF28*	Reduced susceptibility to nucleoside analogues through altered kinase or polymerase activity	Chou (2017) Park et al. (2022)
Human herpesvirus 6	Antiviral activation/target alteration	*U69*, *U38*	Reduced susceptibility through altered viral kinase/polymerase activity	Park et al. (2022)

## 3 Evolution of nanomedicine in AMR therapy

Nanomedicine has evolved from a concept centered on nanoscale drug carriers into an advanced therapeutic platform for precision antimicrobial intervention, owing to the ability of nanoscale materials to improve drug solubility, stability, bioavailability, targeted delivery, and therapeutic efficacy (Huang R. et al., 2024; Ioannou et al., 2024). Early nanoparticles primarily focused on improving pharmacokinetics, while subsequent developments have increasingly emphasized nanoparticle composition, size, morphology, surface chemistry, drug-loading strategies, and responsiveness to biological stimuli to overcome biological barriers, systemic toxicity, and translational challenges.

The evolution of nanomedicine in antimicrobial therapy included early clinically translated lipid-based formulations of amphotericin B, such as AmBisome®, ABELCET®, and Amphotec®, which established an early benchmark for clinical translation due to reduced toxicity and improved therapeutic efficacy compared to standard amphotericin B Fungizone® (Clemons and Stevens, 1998; Zhang et al., 2006); this success was followed by the development of liposome-encapsulated antibiotics, including aminoglycosides (Schiffelers et al., 2001). These lipid systems demonstrated the potential of nanoscale formulations to modify drug distribution, circulation time, tissue accumulation, and therapeutic exposure; however, their utility can be influenced by reticuloendothelial system-mediated clearance (Li and Huang, 2009), Lipid oxidation and hydrolysis during storage can compromise bilayer integrity and membrane permeability, potentially resulting in leakage of encapsulated cargo and changes in vesicle size or structure (Payton et al., 2014; Wang et al., 2024). To address reticuloendothelial-mediated clearance, D-self peptide-labeled liposomes have been developed using CD47^−^and SIRP*α*-derived proteins as a surface functionalization strategy, reducing macrophage-mediated uptake and promoting prolonged circulation (Tang et al., 2019). These early advances in lipid-based antimicrobial delivery laid the foundation for modern antimicrobial nanomedicine, leading to the rapid development and diversification of nanocarrier platforms, including liposomes, polymeric nanoparticles, dendrimers, and solid lipid nanoparticles incorporating self-recognition signals. However, their clinical translation is challenged by multistep synthesis, formulation instability, variable surface functionalization, high production costs, storage requirements, and batch-to-batch variability. Despite substantial preclinical development, the clinical translation of antimicrobial nanomedicines remains limited. Lipid-based inhalable formulations represent important translational successes, particularly amikacin liposome inhalation suspension, which has undergone clinical evaluation for pulmonary nontuberculous mycobacterial infections. The ARISE study and a subsequent 12-month open-label extension supported its clinical evaluation in *Mycobacterium avium* complex lung disease (Daley et al., 2026), while (Ahmed R. et al., 2025) highlighted its translational significance for pulmonary antimicrobial delivery. In contrast, inhaled liposomal ciprofloxacin showed inconsistent outcomes across the phase III ORBIT-3 and ORBIT-4 trials for chronic *P*. *aeruginosa* infection, illustrating the challenges of translating promising nanocarrier systems into reproducible clinical efficacy (Haworth et al., 2019). Overall, clinical translation remains constrained by formulation and manufacturing complexity, pharmacokinetic and biodistribution variability, and the need for robust clinical validation.

During the 2000s and 2010s, metallic nanoparticles such as silver, gold, zinc oxide, and copper oxide (CuO) gained importance because of their intrinsic antimicrobial and antibiofilm properties, offering multi-target mechanisms that may reduce the likelihood of resistance development (Pelgrift and Friedman, 2013; Rai et al., 2009). Their antimicrobial activity is influenced by physicochemical properties such as particle size, surface area, surface charge, morphology, aggregation state, and metal-ion release, which collectively determine their interactions with microbial cells and biological components (Joudeh and Linke, 2022). In biofilms, these physicochemical properties can additionally influence nanoparticle transport, retention, and association with the biofilm matrix and bacterial cells (Fulaz et al., 2019). Particle size influences nanoparticle transport and penetration within biofilms, and larger particles can experience greater steric hindrance and restricted diffusion within the biofilm matrix, as demonstrated for nanoparticles in *Burkholderia multivorans* and *P. aeruginosa* biofilms (Forier et al., 2014). Nanoparticles can interact with microbial cell envelopes through electrostatic, hydrophobic, and other surface-mediated interactions, which can influence membrane association, permeability, and antimicrobial delivery (Huang Y. et al., 2024). These nanoparticles can exert multi-target effects through direct physical interactions with cell membranes and intracellular structures, generation of reactive oxygen species (ROS), disruption of electron transport and membrane integrity, interference with efflux pumps and protein synthesis, and damage to DNA, proteins, and other cellular components (Franco et al., 2022). Metal-based nanomaterials may additionally generate ROS through redox reactions or metal-ion release, causing oxidative damage to membrane lipids, proteins, and nucleic acids (Khleifat et al., 2022; Mishra et al., 2024). Surface charge strongly influences nanoparticle–biofilm interactions through electrostatic forces. The overall charge of the biofilm matrix and its EPS components can influence nanoparticle retention and transport, with electrostatic and hydrophobic interactions contributing to nanoparticle–biofilm association (Fulaz et al., 2019). Despite their potent broad-spectrum activity, their translational potential for systemic applications remains restricted due to high surface reactivity, uncontrolled ion release, aggregation, and long-term persistence, which may contribute to host cytotoxicity and environmental accumulation.

Subsequently, carbon-based nanomaterials, nanogels, graphene oxide, nanoemulsions, and mesoporous silica nanoparticles were developed to enhance targeted drug delivery and biofilm penetration (Dizaj et al., 2015; Huh and Kwon, 2011). Recently, the field has evolved toward multifunctional and stimuli-responsive nanosystems capable of co-delivering antibiotics, antimicrobial peptides, nucleic acids, and CRISPR-Cas components for precision antimicrobial therapy (Ahmed M. E. et al., 2025; Baptista et al., 2018; Mehta et al., 2023). Successful translation requires reproducible control of critical physicochemical attributes, including particle size distribution, polydispersity, surface charge, morphology, drug-loading and encapsulation efficiency, release kinetics, surface-ligand density, and chemical and physical stability. *In vitro* and *in vivo*.

Concurrently, the field has expanded toward the development of biogenic nanoparticles synthesized through green routes using plant extracts and microorganisms. Compared with conventional physicochemical methods, these environmentally sustainable approaches offer improved biocompatibility, reduced toxicity, and potent antimicrobial activity against MDR pathogens through membrane disruption, oxidative stress induction, biofilm inhibition, and enhanced antibiotic delivery, making them suitable for next-generation antimicrobial nanomedicines (Abdallah et al., 2026; Gour and Jain, 2019; Jadoun et al., 2021). However, variability in biological starting materials can affect nanoparticle size, morphology, surface chemistry, composition, and batch-to-batch reproducibility, highlighting the need for standardized synthesis processes.

## 4 Green biogenic synthesis of antimicrobial nanomedicine

Biological resources, including plants, bacteria, fungi, algae, yeasts, and their metabolites, serve as natural reducing, stabilizing, and capping agents for nanoparticle production (Fahim et al., 2024; Yugay and Shkryl, 2026). This green biogenic pathway generally involves the reduction of metal ions using biological metabolites (Arya et al., 2024), such as phytochemicals, proteins, enzymes (reductases), polysaccharides, exopolysaccharides, phenolics, flavonoids, terpenoids, organic acids, *etc.* containing hydroxyl, carboxyl, amino, carbonyl, sulfhydryl, and phosphate functional groups that facilitate metal ion reduction, nanoparticle nucleation, and stabilization (Akdaşçi et al., 2025; Arya et al., 2024). The resulting nanoparticles are subsequently purified through centrifugation, washing, filtration, dialysis, or lyophilization to remove residual reactants and impurities (Bernardes et al., 2025). Following purification, the physicochemical properties of the nanoparticles are characterized using analytical techniques. UV-Vis and FT-IR spectroscopic techniques are employed to evaluate nanoparticle formation and surface functional groups, whereas energy-dispersive X-ray spectroscopy is used to determine elemental composition. Structural characterization is performed using X-ray diffraction to determine crystallinity and phase purity, whereas scanning and transmission electron microscopy are used to assess particle morphology and size. In addition, dynamic light scattering and zeta-potential analysis provide information on particle size distribution, surface charge, and colloidal stability (Chatterjee et al., 2024; Habtemariam et al., 2026; Huq et al., 2023). Collectively, these techniques provide critical insights into nanoparticle physicochemical properties that strongly influence antimicrobial performance, as smaller and highly crystalline nanoparticles typically exhibit enhanced interactions with microbial membranes, increased reactive oxygen species generation, and improved antimicrobial efficacy (U Din et al., 2024; Ravi et al., 2024).

Green-synthesized silver nanoparticles (AgNPs) produced using *Pseudomonas* sp. metabolites exhibited potent antibacterial and antifungal activity through membrane disruption and biofilm inhibition (Plokhovska et al., 2025). Advancing beyond simple monometallic synthesis, recent strategies have achieved the biogenic fabrication of complex multi-component structures. For instance, a green-synthesized CuO nanoparticle/Zn–Al layered double hydroxide nanocomposite produced using *Micromonospora* sp. cell-free supernatant exhibited enhanced antimicrobial activity against several bacterial pathogens, including *Listeria monocytogenes*, demonstrating superior antimicrobial efficacy compared with either CuO nanoparticles or Zn–Al layered double hydroxide alone (Eweis et al., 2024). Furthermore, biogenic cobalt oxide (CoO) and zinc oxide (ZnO) nanoparticles synthesized using rosemary (*Rosmarinus officinalis*) extract exhibited synergistic antimicrobial activity against bacterial pathogens, highlighting the potential of plant-mediated metal oxide nanomaterials as alternative therapeutic agents for combating antimicrobial resistance (Habeeb et al., 2024). Likewise, selenium nanoparticles (SeNPs) are biosynthesized using lactic acid bacterial systems. *Lactiplantibacillus plantarum*-derived SeNPs significantly inhibited MRSA, whereas SeNPs biosynthesized using *Limosilactobacillus fermentum* exhibited strong activity against *C. albicans* with favourable biocompatibility profiles (Kim et al., 2025; Mohamed and El-Zahed, 2024).

Although research on green nanomedicine against viral pathogens remains relatively limited, several biogenic nanoparticles have demonstrated promising antiviral activity. Green-synthesized Cu_2_O, ZnO and selenium-based nanoparticles have demonstrated antiviral activity against SARS-CoV-2 in cell-culture models by disrupting viral attachment, entry, and replication processes (Asmat-Campos et al., 2023; El-Zahed et al., 2026). Collectively, these findings demonstrate that biogenic nanoparticles are transitioning from simple laboratory observations to highly sophisticated, multi-target biomedical platforms uniquely equipped to circumvent established clinical resistance mechanisms. They represent versatile antimicrobial platforms capable of targeting bacterial, fungal, and viral pathogens through multiple complementary mechanisms (Figure 2). Their enhanced biocompatibility and broad-spectrum antimicrobial activity position them as promising candidates for next-generation antimicrobial nanomedicines.

**FIGURE 2:**
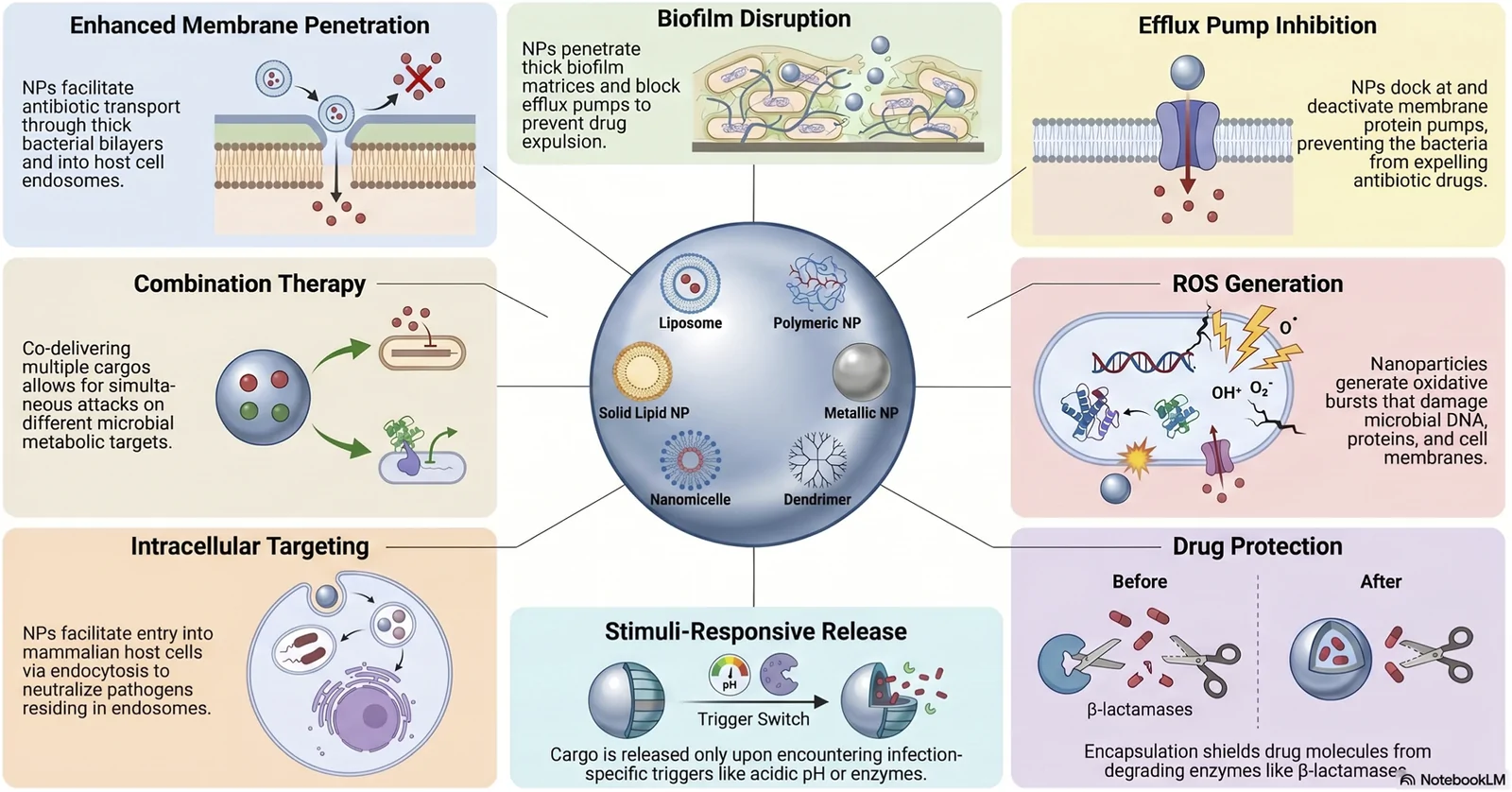
Nanomedicine-mediated mechanisms for overcoming antimicrobial resistance. Nanoparticles improve therapeutic outcomes through enhanced membrane penetration, biofilm disruption, efflux pump inhibition, reactive oxygen species generation, protection of drugs from enzymatic degradation, stimuli-responsive drug release, intracellular targeting, and combination therapy via co-delivery of multiple agents.

## 5 Nanomedicine-antimicrobial synergism

Nanomedicine-antimicrobial synergism has emerged as a promising strategy for addressing resistance by improving drug efficacy, drug delivery, restoring susceptibility in resistant pathogens, and reducing required therapeutic doses (Dove et al., 2023). Metal-based nanoparticles represent some of the most extensively investigated systems and have demonstrated synergistic activity with conventional antimicrobials against drug-resistant bacteria, fungi, and viruses (Table 2). Polymeric, lipid-based and hybrid nanocarrier systems further expand the therapeutic scope by improving drug stability, controlled release, and targeted delivery (Harini and Perumal, 2025). Chitosan-based and lipid-based nanosystems have shown particular promise against drug-resistant bacterial and fungal biofilms by facilitating deeper penetration into microbial communities, prolonging local drug retention, and reducing systemic toxicity (Gamil et al., 2024; Zareshahrabadi et al., 2022; Zomorodian et al., 2023). Chen et al. (2023) developed actively targeted, pH-sensitive curcumin-loaded clustered nanoparticles (anti-CD54@Cur-DA NPs), in which acidic conditions induced charge reversal and particle-size reduction, thereby facilitating biofilm penetration. Compared with free curcumin, the nanoplatform showed stronger inhibition of quorum sensing and greater suppression of biofilm architecture and maturation, accompanied by downregulation of efflux-pump-related genes and enhanced bactericidal activity of penicillin G, ciprofloxacin, and tobramycin. Beyond direct antimicrobial effects, nanoplatforms can modulate innate immune responses and enhance pathogen clearance; Zhu et al. (2024) showed that a multimodal nanoparticle system enhanced neutrophil recruitment and respiratory-burst activity, improving host-mediated killing of antibiotic-resistant *S. aureus* persisters and biofilms. Stimuli-responsive nanoplatforms additionally enable site-specific drug release under pH, enzymatic, or redox triggers, thereby improving therapeutic precision.

**TABLE 2:** Nanomedicine–antimicrobial combinations, mechanisms, therapeutic outcomes, and translational status against infectious pathogens.

Nanoplatform	Antimicrobial	MDR pathogen(s)	Key mechanism	Outcome	Clinical development stage	Toxicity/limitations	Regulatory status	Evidence level	References
AgNPs	Vancomycin	Vancomycin-resistant *E. faecium* (VRE)	Cell-wall destabilization, oxidative stress, enhanced antibiotic localization/penetration, and DNA deformation	MIC reduced from 13.5 to 3.38 mg/L for AgNPs and from 512 to 2 mg/L for vancomycin; FIC = 0.25	Preclinical	No significant cytotoxicity to HaCaT keratinocytes at tested concentrations; further *in vivo* safety validation required	Not established for this formulation	*In vitro*	Válková et al. (2025)
HA-P3-Lipo	Vancomycin	MRSA	Enzyme-responsive HA targeting, TLR4 targeting, and sustained vancomycin delivery	2-fold higher antibacterial activity and 5-fold greater biofilm inhibition than free vancomycin; 76% vancomycin release over 48 h	Preclinical	Biocompatibility reported; further safety and translational validation required	Not established for this formulation/indication	*In vivo*	Ismail et al. (2024)
PEtOx/ZnO NPs	Ciprofloxacin	*S. aureus*, *P. aeruginosa*	ZnO-associated enhancement of antibacterial activity and nanoparticle-mediated drug delivery	Enhanced bactericidal activity; quantitative comparative outcome not reported	Preclinical	Cytotoxicity was cell-type dependent; further *in vivo* validation required	Not established	*In vitro*	Sabuj et al. (2023)
Liposomal ciprofloxacin	Ciprofloxacin	Chronic *P. aeruginosa* infection	Liposomal encapsulation for localized pulmonary delivery and sustained antibiotic exposure	ORBIT-4 significantly prolonged time to first pulmonary exacerbation, whereas ORBIT-3 and the pooled analysis did not show significant benefit	Phase III	Inconsistent efficacy across replicate phase III trials; further evaluation required	Not established	Clinical	Haworth et al. (2019)
CIP@Ag NPs	Ciprofloxacin	*S. aureus*, *B. subtilis*, *P. aeruginosa*, *K. pneumoniae*, *E. coli*	AgNP-mediated membrane disruption/ROS generation with enhanced ciprofloxacin delivery	Zone of inhibition at 5 μg/mL: 31 ± 1.41 mm (*S. aureus*), 33.5 ± 0.7 mm (*P. aeruginosa*), 34 ± 1.41 mm (*B. subtilis*), 34 ± 1.41 mm (*K. pneumoniae*), and 30.0 ± 0.9 mm (*E. coli*)	Preclinical	Further *in vivo* safety and efficacy validation required	Not established	*In vitro*	Laib et al. (2025)
Col/haNPs	Colistin	MDR *A. baumannii* and carbapenem-resistant *K. pneumoniae*	Prolonged colistin release and enhanced antibiotic delivery	MIC decreased to 1.25–2.5 μg/mL for resistant isolates; antibiofilm activity also reported (*p <* 0.05)	Preclinical	Negligible cytotoxicity in human fibroblasts and low hemolytic activity; further *in vivo* safety validation required	Not established	*In vitro*	Scutera et al. (2021)
COL-NE	Colistin	MDR *A. baumannii*	Enhanced intracellular delivery/penetration and improved colistin delivery	27%–45% lower *in vitro* toxicity than free colistin; lung bacterial burden reduced by 2 log_10_ CFU/mL versus untreated mice	Preclinical	Further long-term safety, pharmacokinetic and translational validation required	Not established	*In vitro* and *in vivo*	Martínez-Guitián et al. (2026)
NTZ-AuNPs	Nitazoxanide	CRE	Membrane disruption, increased permeability, ROS generation, and impaired ATP synthesis	Bactericidal activity, reduced bacterial burden, and improved survival in mice; quantitative values not reported in the table source	Preclinical	Favorable biocompatibility reported; further translational validation required	Not established	*In vivo*	Yao et al. (2026)
Tet-CPNPs	Tetracycline	Drug-resistant *E. coli*, *Shigella flexneri*, *Salmonella kentucky*	Enhanced cellular penetration and delivery of tetracycline	Approximately 4-log_10_ fold reduction in bacterial colonization	Preclinical	No major toxicity reported; further comprehensive safety validation required	Not established	*In vitro* and *in vivo*	Mukherjee et al. (2019)
C7-3 peptide-loaded CNPs	C7-3 peptide and derivatives	MDR *N. gonorrhoeae*	Chitosan-mediated membrane interaction and peptide delivery; proposed AniA inhibition	MIC/MIC_50_ = 3.44 μg/mL; ∼40% gonococcal inhibition and 68.8% biofilm inhibition at the reported concentrations	Preclinical	Cytocompatibility demonstrated in HeLa cells; further *in vivo* validation required	Not established	*In vitro*	Albdrawy et al. (2024)
IONPs-CS-MCZ	Miconazole	*C. albicans*, *C. glabrata*	Enhanced cellular-level delivery of miconazole and synergistic antifungal activity	Lower MIC than free miconazole; reduced *C. albicans* biofilm CFU and metabolic activity	Preclinical	Further safety and *in vivo* validation required	Not established	*In vitro*	Arias et al. (2020)
CS-MCZ	Miconazole	*C. albicans*	Chitosan-mediated mucoadhesion and enhanced local delivery	Significant reduction in *Candida* CFU (*p <* 0.0001) with improved clinical signs and symptoms	Randomized controlled trial	No adverse reactions reported during the 28-day study; longer-term safety and efficacy require further validation	Not established for this nanoparticle formulation	Clinical/*in vivo*	Gamil et al. (2024)
Biogenic AgNPs	Fluconazole; metronidazole	*C. albicans*	Synergistic interaction between AgNPs and antifungal agents	FIC = 0.70; synergistic antifungal activity at lower concentrations	Preclinical	Mammalian-cell toxicity not comprehensively evaluated; further safety and *in vivo* validation required	Not established	*In vitro*	Rozhin et al. (2024)
Col-SeNPs	Colistin	MDR *P. aeruginosa*; resistant *Candida* spp.	*mexY* downregulation, membrane permeabilization, and oxidative stress	MIC = 125 μg/mL for reported antibacterial activity; antifungal activity significant (*p <* 0.001)	Preclinical	Dose-dependent cytotoxicity observed in MCF-7 cells; further *in vivo* safety and pharmacokinetic validation required	Not established	*In vitro*	Ahmed M. E. et al. (2025)
Se@TP	β-Thujaplicin	H1N1 influenza virus	Suppression of ROS and caspase-3-mediated apoptosis	65% survival in infected mice	Preclinical	Lower toxicity reported; further safety and translational validation required	Not established	*In vitro* and *in vivo*	Wang et al. (2020)
Ribavirin-loaded mPEG-PCL MNPs	Ribavirin	Zika virus	pH-responsive micelle dissociation and intracellular ribavirin release	EC_50_ = 0.2 nM; approximately 1,000-fold lower ribavirin concentration required than free ribavirin	Preclinical	No cytotoxicity observed at tested concentration; further *in vivo* safety and efficacy validation required	Not established	*In vitro*	Blahove et al. (2024)
EFV-NMs	Efavirenz	DENV-2	Enhanced aqueous solubility and micellar delivery	1.8- And 4.7-log reductions in viral burden in A549 and Vero cells, respectively	Preclinical	Good *in vitro* cytocompatibility and no gastrointestinal tissue damage in rats; antiviral efficacy in animal models remains to be established	Not established	*In vitro* and *in vivo*	Maldonado et al. (2025)
Ag/ZnO/Amodiaquine	Amodiaquine	SARS-CoV-2, H1N1, HSV	Proposed viral-surface disruption and possible ROS-mediated effects	>7-log SARS-CoV-2 reduction and >6-log HSV/H1N1 reduction within 30 s; symptom improvement reported in exploratory clinical study	Clinical (exploratory)	No toxicity at tested concentration; further controlled clinical and safety validation required	Not established	*In vitro and* *in vivo*	Dolatyari and Rostami (2022)
CNC@Pyc.MOL.ZnO	Molnupiravir	Human coronavirus 229E	pH-dependent molnupiravir release and enhanced drug delivery	37.6% viral inhibition at 800 μg/mL	Preclinical	High cell viability reported; clinical trials required	Not established	*In vitro*	El-Shafai et al. (2024)
SNS812	SNS812 siRNA	SARS-CoV-2	RNA interference targeting conserved RdRp	Favorable safety and tolerability in first-in-human phase I study; quantitative antiviral efficacy not established	Phase I	No major safety concerns reported; further efficacy validation required	Not established	Clinical	Chang et al. (2025)
MIR 19® siRNA–peptide dendrimer	SARS-CoV-2-specific siRNA	SARS-CoV-2	RNA interference targeting conserved RdRp sequence	Median time to clinical improvement: 6 days versus 8 days in controls at the low dose; viral load also significantly reduced	Phase II	No treatment-related adverse events identified; high-dose group did not show significant clinical efficacy	Not established	Clinical	Khaitov et al. (2023)

Abbreviations: AgNPs, silver nanoparticles; AuNPs, gold nanoparticles; CFU, colony-forming units; CIP@Ag NPs, ciprofloxacin-loaded silver nanoparticles; CNC, cellulose nanocrystals; COL-NE, colistin-loaded nanoemulsion; CNPs, chitosan nanoparticles; CRAB, carbapenem-resistant *A*. *baumannii*; CRE, carbapenem-resistant Enterobacteriaceae; CS-MCZ, miconazole-loaded chitosan nanoparticles; DENV-2, dengue virus serotype 2; EC_50_, half-maximal effective concentration; EFV-NMs, efavirenz-loaded nanomicelles; HA, hyaluronic acid; HA-P3-Lipo, hyaluronic acid-coated, TLR4-targeting peptide-functionalized liposomes; HSV, herpes simplex virus; IONPs-CS-MCZ, miconazole-loaded chitosan-coated iron oxide nanoparticles; MDR, multidrug-resistant; MIC, minimum inhibitory concentration; MNPs, micellar nanoparticles; MRSA, methicillin-resistant *Staphylococcus aureus*; N/Se@TCsNPs, nisin/selenium-loaded thiolated chitosan nanoparticles; NPs, nanoparticles; NTZ-AuNPs, nitazoxanide-functionalized gold nanoparticles; PEtOx, poly (2-ethyl-2-oxazoline); PQA-Az-13, polycationic quinolone analogue Az-13; RdRp, RNA-dependent RNA, polymerase; ROS, reactive oxygen species; siRNA, small interfering RNA; TLR4, Toll-like receptor 4; VRE, vancomycin-resistant *E*. *faecium*; VL-AuNPs, virstatin-conjugated gold nanoparticles.

The evidences summarized in Table 2 shows that the magnitude of reported benefits varies substantially across formulations and experimental models. Some systems reported substantial reductions in MIC, bacterial burden, biofilm formation, or viral replication, whereas others showed more modest improvements. However, these numerical outcomes cannot be directly compared because the studies differ considerably in pathogen strains, nanoparticle composition, drug concentrations, exposure times, assay methods, and efficacy endpoints. Thus, large quantitative effects observed under experimental conditions should be interpreted in the context of model-specific limitations rather than assumed to predict equivalent clinical benefits.

The same nanomedicine principles are increasingly being applied to antiviral therapy, although viral infections present distinct delivery challenges. Metallic, polymeric, and hybrid nanoplatforms have demonstrated improved efficacy against diverse viral pathogens, including respiratory and flaviviral infections, by reducing viral load and limiting cytopathic effects (Dolatyari and Rostami, 2022; El-Shafai et al., 2024). However, effective antiviral delivery requires overcoming tissue and mucosal barriers, achieving cellular uptake, avoiding endosomal sequestration and intracellular degradation, and depending on the target, delivering therapeutic to the cytoplasm or nucleus. These requirements are particularly important for RNA therapeutics, for which protection from extracellular degradation, efficient cellular uptake, endosomal escape, and intracellular release are critical determinants of efficacy.

Lipid nanoparticles (LNPs) have emerged as an important platform for addressing these intracellular delivery barriers because their lipid composition can be engineered to protect RNA cargo, facilitate cellular uptake, and promote endosomal escape and cytosolic RNA release (Zheng et al., 2023). Intranasal administration of LNP-formulated siRNAs reduced SARS-CoV-2 and respiratory syncytial virus infection *in vivo*, whereas naked siRNA showed limited antiviral activity, highlighting the importance of nanocarrier-mediated delivery (Supramaniam et al., 2023). Similarly, LNP-encapsulated delivery of siRNAs targeting host factors such as DOCK11 and deubiquitinase USP33 has demonstrated antiviral effects in experimental models of hepatitis B virus and SARS-CoV-2 infection, respectively (Okada et al., 2024; Zhou et al., 2024). These findings highlight that effective RNA delivery depends not only on cargo protection and nanoparticle accumulation but also on efficient cellular uptake, endosomal escape, and cytosolic release. Accordingly, next-generation antiviral LNPs require coordinated optimization of tissue targeting, cellular uptake, endosomal escape, RNA stability, and intracellular release rather than simply maximizing nanoparticle accumulation.

Clinical translation of RNA nanotherapeutics has also progressed beyond preclinical studies. Chang et al. (2025) reported a first-in-human phase I study of the inhaled, fully modified siRNA SNS812, which targets the conserved RNA-dependent RNA polymerase of SARS-CoV-2 and demonstrated favorable safety and tolerability. In a separate phase II randomized controlled trial, the inhaled SARS-CoV-2-specific siRNA–peptide dendrimer formulation MIR 19® (siR-7-EM/KK-46) significantly reduced the time to clinical improvement at the low dose in hospitalized patients with moderate COVID-19 (Khaitov et al., 2023). Nevertheless, clinical translation of antiviral nanomedicine remains more limited than its preclinical development, with challenges related to virus-specific targeting, tissue distribution, pharmacokinetics, immunogenicity, long-term safety, and scalable manufacturing. Thus, although LNP-based RNA technologies provide an important translational foundation, their broader application to antiviral therapy will require reproducible manufacturing and dosing strategies, improved tissue-selective delivery, efficient endosomal escape, and control of unwanted immune responses.

A similar translational gap is evident across antibacterial and antifungal nanomedicine. Most formulations in Table 2 remain at the *in vitro* or preclinical stage, despite frequently reporting substantial improvements in antimicrobial activity. Importantly, progression to clinical testing has not consistently resulted in superior therapeutic outcomes. Inhaled liposomal ciprofloxacin, produced discordant results in the phase III ORBIT-3 and ORBIT-4 trials, with a significant benefit observed in ORBIT-4 but not ORBIT-3 or the pooled analysis (Haworth et al., 2019). Conversely, miconazole-loaded chitosan nanoparticles have progressed to a randomized clinical study for oral candidiasis, illustrating that some nanocarrier systems have moved beyond preclinical evaluation, although longer-term efficacy and safety remain to be established.

An additional issue that warrants greater attention is the possibility that microorganisms may adapt to nanoparticle exposure. The broad and multi-targeted activity of nanomaterials is often considered advantageous for reducing the likelihood of resistance; however, prolonged or sublethal exposure may impose selective pressure that favors less-susceptible populations. Experimental evolution of *A*. *baumannii* demonstrated the development of resistance to silver nanoparticles following prolonged exposure, accompanied by mutations associated with cell-surface attachment and capsular polysaccharide synthesis, altered biofilm growth, and enhanced protection against oxidative stress (McNeilly et al., 2023). Exposure to silver nanoparticles has also been reported to alter biofilm community structure and the abundance of antibiotic-resistance genes (Bao et al., 2023). Metal exposure may also promote co-selection of metal and antibiotic resistance under shared selective pressures (Heydari et al., 2023). Together, these findings challenge the assumption that the multi-targeted activity of nanomaterials inherently limits resistance development and indicate that resistance evolution should be evaluated alongside efficacy during long-term development and use.

Overall, nanomedicine–antimicrobial synergism offers a versatile approach for enhancing conventional antimicrobial therapies through improved delivery, controlled release, intracellular targeting, and complementary mechanisms of microbial inhibition. However, the therapeutic potential of these systems should be considered alongside unresolved issues of resistance evolution, toxicity, pharmacokinetics, manufacturing, and clinical reproducibility. Future research should therefore move beyond demonstrating short-term increases in antimicrobial activity toward defining the mechanisms, exposure conditions, resistance trajectories, and delivery parameters that determine whether nanomedicine–antimicrobial combinations provide durable clinical benefits against difficult-to-treat bacterial, fungal, and viral infections.

## 6 Next-generation antimicrobial nanomedicine platforms

The rapid emergence of MDR pathogens has accelerated the development of next-generation antimicrobial nanomedicine platforms that combine targeted drug delivery, and pathogen-specific activity. Unlike conventional nanocarriers that primarily improve the pharmacokinetics of antimicrobial agents, advanced nanomedicinec systems are designed to actively overcome resistance mechanisms, eliminate intracellular pathogens, and enhance therapeutic precision (Adedoyin et al., 1997; Hayat et al., 2025; Obeid et al., 2025; Parvin et al., 2025). Biomimetic cell membrane-coated nanoparticles, which utilize natural cell membranes derived from erythrocytes, macrophages, neutrophils, platelets, *etc.*, enabling prolonged circulation, immune evasion, and improved interaction with pathogenic bacteria, are the most promising approaches (Song et al., 2023), that also improve antibiotic delivery while reducing systemic toxicity and inflammatory damage. In addition, bacterial membrane-coated nanoparticles can function as nanovaccines by stimulating pathogen-specific immune responses, thereby providing both therapeutic and preventive benefits (Fu et al., 2025; Sun et al., 2022). Similarly, nanostructured antimicrobial peptide delivery systems represent another transformative platform for combating resistant pathogens. Although antimicrobial peptide exhibits broad-spectrum antimicrobial activity and a low tendency for resistance development, their clinical application is limited by enzymatic degradation and short half-life (Fadaka et al., 2021). Nanomedicine-based antimicrobial peptide encapsulation within liposomes-, polymeric nanoparticles-, dendrimers-based formulations have shown remarkable efficacy against MDR Gram-negative bacteria by improving membrane penetration and protecting peptides from proteolytic degradation (Saleem et al., 2026; Yang et al., 2021). Nanoparticle-mediated delivery enables CRISPR components to selectively eliminate resistance genes, disrupt biofilm formation, and induce bacterial cell death while minimizing off-target effects on beneficial microbiota; however, efficient intracellular delivery and large-scale clinical translation remain major challenges (Mayorga-Ramos et al., 2023; Wizrah, 2026).

Metal-organic framework (MOFs) can encapsulate antibiotics and antimicrobial peptides while providing stimuli-responsive drug release triggered by pH, redox conditions, or enzymatic activity (Chandra et al., 2025). Hybrid MOF nanocomposites integrating photothermal, photodynamic, and catalytic functionalities have demonstrated enhanced antimicrobial efficacy against intracellular and biofilm-associated infections (Lu et al., 2026; Qi et al., 2023). Alongside these emerging platforms, advanced antimicrobial drug-delivery systems including liposomes, polymeric micelles, nanogels, nanoemulsions, solid lipid nanoparticles, and metallic nanoparticles, have significantly expanded the therapeutic scope of nanomedicine (Alqarni et al., 2022). These systems improve drug stability, bioavailability, intracellular delivery, and biofilm penetration while enhancing the efficacy of antibiotic, antifungal, and antiviral agents against resistant microbial pathogens (Pu et al., 2025; Zong et al., 2022). These smart systems respond to infection-associated microenvironmental cues, enabling controlled targeted drug release minimizing off-target toxicity, enhancing biofilm penetration, improving antimicrobial efficacy, and reducing the likelihood of resistance development (Sousa et al., 2023). Collectively, next-generation antimicrobial nanomedicine platforms are transforming infection management by integrating biomimetic camouflage, antimicrobials delivery, multifunctional nanocarriers and stimuli-responsive release. Despite their sophisticated design and promising *in vitro* performance, translating advanced nanoplatforms into clinical settings remains challenging because of complex pharmacological and manufacturing requirements. Following systemic administration, nanoparticles rapidly interact with plasma proteins and develop a dynamic protein corona that can alter surface-ligand accessibility, cellular uptake, biodistribution, and targeting performance (Chou and Lin, 2024; Kim et al., 2023). Predictable pharmacokinetic and biodistribution profiles also remain difficult to achieve because of clearance by the mononuclear phagocyte system and interindividual physiological variability (Cisneros et al., 2024). From a manufacturing perspective, translating multicomponent biomimetic, hybrid, and stimuli-responsive systems from laboratory-scale production to Good Manufacturing Practice-compliant manufacturing requires stringent control of critical quality attributes, structural integrity, batch-to-batch reproducibility, scalability, and production costs. These challenges highlight the need to integrate pharmacokinetic, biodistribution, manufacturing, and commercialization considerations into nanoparticle design at an early stage of development. A comparative overview of conventional antimicrobial therapies and nanomedicine-based approaches across key mechanistic, therapeutic, and translational parameters is presented in Table 3, highlighting both the advantages and current limitations of nanomedicine.

**TABLE 3:** Traditional antibiotics and nanomedicine: a comparative framework of mechanistic targets, resistance risk, and therapeutic efficiency.

Parameter	Traditional antimicrobials	Nanomedicine-based antimicrobials
Therapeutic target	Specific molecular targets such as cell wall synthesis, ribosomes, nucleic acid synthesis, sterol biosynthesis, or viral polymerases	Multiple targets including microbial membranes, intracellular organelles, biofilms, nucleic acids, and infection microenvironment
Mechanism(s)	Usually single-target or limited mechanism	Multifactorial; combines drug delivery, membrane disruption, ROS generation, photothermal/photodynamic killing, catalytic activity, and immune modulation
Activity against MDR/XDR	Frequently reduced due to established resistance mechanisms	Often enhanced because nanoparticles have multi-target activity to disrupt resistance mechanisms
Enzymatic drug degradation	Highly susceptible to *β-*lactamases, aminoglycoside-modifying enzymes, and other drug-inactivating enzymes	Reduced impact due to nanoparticle-mediated drug shielding and intracellular delivery
Target-site modification	Major cause of therapeutic failure	Less affected because nanomedicine may act through multiple simultaneous mechanisms
Efflux pump susceptibility	High; drugs may be actively expelled by ABC, MFS, RND, MATE, and SMR transporters	Reduced due to enhanced intracellular accumulation and membrane penetration
Membrane permeability	Strongly affected by porin loss, membrane remodelling, and fungal sterol changes	Nanoparticles can improve penetration through bacterial/fungal membranes
Biofilm penetration	Often poor due to extracellular matrix barrier	Enhanced penetration and disruption of biofilm architecture
Intracellular pathogen targeting	Limited intracellular delivery	High potential for targeted intracellular delivery
Target specificity	Can be broad-spectrum	Can be broad-spectrum or engineered for site-specific
Resistance development risk	High due to mutation, gene transfer, and selection pressure	Lower but emerging adaptive resistance to nanoparticles has been reported
Mechanisms of resistance development	Enzymatic degradation, target alteration, efflux, biofilm formation	Membrane remodelling, reduced uptake, nanoparticle aggregation, oxidative stress adaptation, metal efflux
Diagnostic integration	Minimal	Possible through theragnostic nanoplatforms with imaging capability
Toxicity concerns	Organ toxicity, nephrotoxicity, hepatotoxicity, microbiome dysbiosis*etc.*	Nanotoxicity, ROS-mediated cytotoxicity, organ accumulation, microbiome dysbiosis*etc.*
Environmental concerns	Drug residues may promote AMR in environment	Nanoparticle persistence, bioaccumulation, ecological toxicity
Manufacturing complexity	Established and scalable	Complex synthesis, characterization, and batch reproducibility challenges
Clinical translation	Widely approved and standardized	Mostly preclinical or early clinical stage
Development cost	Generally lower	Higher due to advanced engineering and regulatory requirements

Abbreviations: MDR, multidrug-resistant; XDR, extensively drug-resistant; ROS, reactive oxygen species; ABC, ATP-binding cassette; MFS, major facilitator superfamily; RND, resistance-nodulation-division; MATE, multidrug and toxic compound extrusion; SMR, small multidrug resistance.

## 7 Toxicity and biosafety of nanomedicine

The physicochemical properties that contribute to antimicrobial activity, including small particle size, high surface area, increased reactivity, and enhanced cellular penetration, may also promote unintended interactions with biological systems (Ji et al., 2024). Several studies have demonstrated nanoparticle-associated toxicity including excessive oxidative stress, lipid peroxidation, accumulation in organs, behavioural changes, mitochondrial dysfunction, DNA damage, inflammation, and apoptosis (Egbuna et al., 2021; Fernández-Bertólez et al., 2024; Jalili et al., 2020; Wu et al., 2022; Xuan et al., 2023; Zinicovscaia et al., 2021). Although newer nanoplatforms such as RNA nanoparticles and green-synthesized gold nanoparticles have shown improved biocompatibility profiles, comprehensive long-term safety evaluations remain limited (Aljohani et al., 2022). Beyond direct host toxicity, antimicrobial nanoparticles may also alter microbial diversity, intestinal immune homeostasis, disrupted intestinal immune regulation through microbiota-associated effects, and metabolic functions of beneficial microbial communities (Ma et al., 2023; Ren et al., 2023; Zhang et al., 2022). Furthermore, food-grade metal oxide nanoparticles altered intestinal microbial populations, epithelial morphology, and nutrient absorption functions *in vivo* (Cheng et al., 2023). These findings suggest that prolonged nanoparticle exposure may contribute to microbiome dysbiosis and associated physiological consequences (Sanati et al., 2025). However, bacteria can reduce nanoparticle susceptibility through genetic mutations, metal efflux pump upregulation, structural remodeling (membrane rigidification, cell wall alterations) decreased uptake, oxidative stress adaptation, membrane modification, *etc*., (Hochvaldová et al., 2024; Kamat and Kumari, 2023; Niño-Martínez et al., 2019). In addition, resistance determinants associated with metal tolerance may coexist with antibiotic resistance genes, potentially facilitating co-selection of antimicrobial resistance (Elbehiry et al., 2022).

The biosafety concerns of nanomedicine extend beyond human health to encompass broader environmental and ecological systems. Following production, clinical application, and disposal, nanoparticles may be released into sewage and enter in terrestrial and aquatic environments, where they can undergo complex processes including transport, transformation, aggregation, dissolution, sedimentation, and bioaccumulation (Martinez et al., 2020; Mishra and Sundaram, 2023). Environmental exposure to engineered nanomaterials has been associated with alterations in microbial community composition, disruption of nutrient cycling, oxidative stress induction, and adverse effects across multiple trophic levels, including microorganisms, plants, invertebrates, and vertebrates (Das and Paul, 2025; Kumah et al., 2023; Martinez et al., 2020; Mishra and Sundaram, 2023; Zapałowska et al., 2026). Moreover, the persistence of nanoparticles in environmental compartments may exert selective pressure on microbial communities and contribute to the emergence of nanoparticle-resistant strains (Figure 3). Overall, while nanomedicine offers significant opportunities for improving antimicrobial therapy, its successful clinical translation requires careful consideration of toxicity, microbiome perturbation, microbial adaptation, and environmental impacts. Continued investigation of nanoparticle biodistribution, long-term safety, and ecological consequences will be essential for the safe and sustainable development of antimicrobial nanomedicines.

**FIGURE 3:**
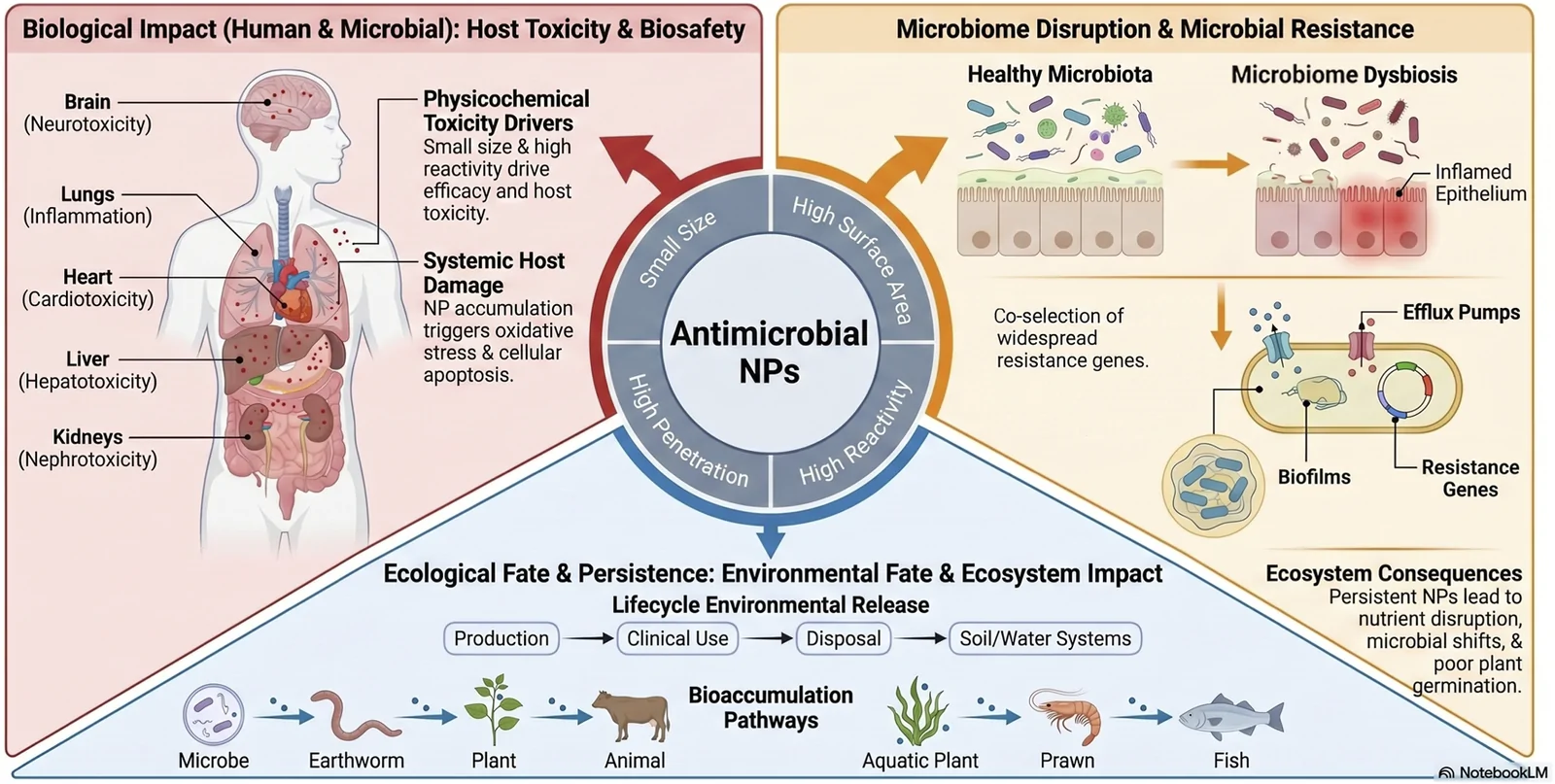
Schematic illustration of the multi-dimensional impact of antimicrobial nanomedicine on host biology, microbial resistance, microbiome stability, and environmental ecosystems.

## 8 Future perspectives and research gaps

Despite remarkable advances in antimicrobial nanomedicine, several scientific and translational barriers must be overcome before these technologies can achieve widespread clinical implementation. Green biogenic synthesis represents a promising route toward sustainable antimicrobial nanomedicine; however, significant challenges remain regarding large-scale manufacturing, quality control, and batch-to-batch reproducibility. Variability in biological feedstocks can substantially influence nanoparticle physicochemical properties and therapeutic performance. Therefore, future efforts should focus on developing standardized production protocols, scalable bioprocessing systems, and regulatory frameworks capable of supporting industrial translation while maintaining consistency and safety.

Future antimicrobial nanomaterials should increasingly be developed under Safe-by-Design principles, where toxicity, biodegradability, biodistribution, and environmental impact are considered during the earliest stages of nanoparticle engineering. An additional challenge is the emerging evidence that microorganisms may develop adaptive responses to nanomaterials following prolonged or sublethal exposure. Although nanoparticles generally possess multitarget antimicrobial mechanisms, resistance-associated traits involving membrane remodelling, oxidative stress adaptation, efflux activity, and biofilm-associated protection have been reported (Hochvaldová et al., 2024; Kamat and Kumari, 2023). Future research should therefore investigate nanoparticle resistance evolution, cross-resistance potential, and the effectiveness of combination therapies designed to minimize selective pressure while maximizing antimicrobial efficacy. Looking ahead, emerging autonomous and stimuli-responsive nanosystems capable of sensing biological signals and adapting therapeutic responses offer opportunities for precision antimicrobial therapy. Their development will increasingly benefit from the integration of computational approaches capable of guiding rational nanomaterial design and optimization.

Machine learning and artificial intelligence-assisted nanomedicine are emerging approaches for accelerating nanoparticle design and optimization by linking physicochemical properties and formulation parameters with biological responses and therapeutic outcomes, thereby reducing reliance on trial-and-error approaches (Agrahari et al., 2024; Gao et al., 2024; Rao et al., 2024; Li, W. R. et al., 2025). Recent studies have demonstrated the feasibility of machine-learning-guided optimization of nanoparticle properties and cellular uptake (Ortiz-Perez et al., 2024; Seegobin et al., 2024). Complementary experimental platforms, such as organ-on-chip models, could further support the translation of precision antimicrobial nanomedicine by providing physiologically relevant systems for evaluating host–pathogen interactions and antimicrobial responses. Kaden et al. (2024) used an intestine-on-chip model to examine *C. albicans* infection and responses to clinically relevant caspofungin exposure. Digital twin technologies may eventually integrate patient-specific clinical, microbiological, and treatment data to model therapeutic responses and support individualized antimicrobial management (Esposito et al., 2025). Together, these approaches could advance precision nanomedicine by enabling pathogen-, infection-site-, and patient-specific selection of nanocarriers and antimicrobial therapies, supporting more personalized antimicrobial treatment. However, these applications remain at an emerging stage, and their clinical utility will depend on reliable datasets and experimental validation. The continued integration of nanotechnology, microbiology, computational approaches, and translational research will be crucial for advancing these emerging strategies toward clinical application.

## 9 Conclusion

The rapid global emergence of antimicrobial resistance AMR in bacterial, fungal, and viral pathogens has reduced the effectiveness of conventional antimicrobial therapies, creating a major therapeutic challenge. Resistance mechanisms such as enzymatic drug inactivation, target modification, efflux-mediated drug extrusion, biofilm formation, and genetic adaptation continue to drive multidrug resistance and treatment failure. Nanomedicine offers a promising strategy to address these challenges through its ability to enhance drug delivery, improve bioavailability, disrupt biofilms, and exert multitarget antimicrobial effects. Green biogenic nanomedicine and nanoparticle-antimicrobial synergism further provide complementary approaches for improving therapeutic efficacy against resistant pathogens, while, biomimetic, CRISPR-based, stimuli-responsive, and theragnostic nanosystems, are expanding the scope of precision antimicrobial therapy. However, successful translation requires careful consideration of careful control of nanoparticle physicochemical properties, formulation reproducibility, toxicity, biosafety, environmental impact, resistance evolution, and clinical translation. Emerging approaches such as AI-assisted nanoparticle design, organ-on-chip models, and patient-specific computational technologies may further support precision and personalized antimicrobial therapy. Addressing these challenges will require integrated mechanistic, computational, experimental, and translational research to advance nanomedicine toward safe, effective, and clinically applicable strategies against AMR.

## Glossary

AAC: Aminoglycoside *N-*acetyltransferase
AdeABC: *Acinetobacter* drug efflux ABC transporter system
AMR: Antimicrobial resistance
APH: Aminoglycoside *O-*phosphotransferase
CMY: Cephamycinase *β-*lactamase
CRISPR: Clustered Regularly Interspaced Short Palindromic Repeats
ESBL: Extended-spectrum *β-*lactamase
HIV: Human immunodeficiency virus
KPC: *Klebsiella pneumoniae* carbapenemase
MDR: Multidrug resistance
MERS-CoV: Middle East respiratory syndrome coronavirus
MFS: Major facilitator superfamily
MRSA: Methicillin-resistant *Staphylococcus aureus*
NPs: Nanoparticles
OmpA/OmpC/OmpF: Outer membrane proteins
OXA: Oxacillinase *β-*lactamase
PhoE: Phosphate porin E
SARS-CoV-2: Severe acute respiratory syndrome coronavirus 2
SHV: Sulfhydryl variable *β-*lactamase
TEM: Temoniera *β-*lactamase
VEB: Vietnamese extended-spectrum *β-*lactamase
VRE: Vancomycin-resistant *Enterococcus*
ZnO: Zinc oxide
ABC: ATP-binding cassette
AgNPs: Silver nanoparticles
ANT: Aminoglycoside *O-*nucleotidyltransferase
AuNPs: Gold nanoparticles
CTX-M: Cefotaximase-Munich *β-*lactamase
CuO: Copper oxide
GES: Guiana extended-spectrum *β-*lactamase
IMP: Imipenemase metallo-*β-*lactamase
MATE: Multidrug and toxic compound extrusion transporter
MdtABC: Multidrug transporter ABC system
MexAB–OprM: Multidrug efflux pump in *P. aeruginosa*
MOF: Metal–organic framework
NDM: New Delhi metallo-*β-*lactamase
Omp: Outer membrane protein
OprD/OprF/OprH: Outer membrane porins
PBP: Penicillin-binding protein
ROS: Reactive oxygen species
SeNPs: Selenium nanoparticles
SMR: Small multidrug resistance transporter
**TiO**_**2**_: Titanium dioxide
VIM: Verona integron-encoded metallo-*β-*lactamase
XDR: Extensively drug-resistant
