# Supplementary material for: Nanomedicine against antimicrobial resistance: mechanistic insights and next-generation therapeutic potential
Source: Front Chem. 2026 Sep 14;14:1915145. doi: 10.3389/fchem.2026.1915145 (PMC13617642; doi:10.3389/fchem.2026.1915145)
Supplement: Supplementary file 1 [file DataSheet1.pdf]

Nanomedicine against antimicrobial resistance: Mechanistic insights and next-generation therapeutic potential

Vishal L. Handa[a], Benedict B. Vyas[b], Dr. Abhishek Padhi[c], Dr. Ashwini Agarwal[c], BRM Vyas[b]

[a]Project Consultant, NOHP-PCZ, National Center for Disease Control, All India Institute of Medical Sciences, Rajkot, Gujarat, India, 360110

[b]Department of Biosciences, Saurashtra University, Rajkot, Gujarat, India, 360005

[c]Department of Clinical Microbiology and Infectious Disease, All India Institute of Medical Sciences, Rajkot, Gujarat, India, 360110

*Corresponding author: Dr. BRM Vyas, Department of Biosciences, Saurashtra University, Rajkot, Gujarat, India, 360005. Email: brmvyas@hotmail.com

Supplementary Material

Figure 1. Major mechanisms of antimicrobial drug resistance in bacteria, fungi, and viruses prompt in ChatGPT 5.5

Prompt: Mechanisms of Drug Resistance in Bacteria, Fungi, and Viruses

Create a publication-quality scientific schematic figure:

General Figure Settings

- **Figure size:** 190 mm (W) × 160 mm (H)
- **Layout:** Three vertical panels arranged left-to-right
  - **Panel A:** Bacterial Cell (largest panel; ~42% width)
  - **Panel B:** Fungal Cell (middle panel; ~28% width)
  - **Panel C:** Viral Resistance (right panel; ~30% width)
- **Background:** White
- **Style:** Scientific vector illustration, clean, flat, publication-quality
- **Font:** Arial or Helvetica
- **Font size:** 8–10 pt

- **Text rule:** Minimal but informative; short mechanistic labels
- **Arrows:** Black solid arrows for active pathways; dashed arrows for inhibited processes
- **Inhibition:** Red “X”
- **Panel header colors:**
    - Panel A: Dark blue
    - Panel B: Dark green
    - Panel C: Dark red
- Use rounded panel borders matching header color.

PANEL A — BACTERIAL CELL

Header label:
A | BACTERIAL CELL

Layout

Show a **cross-sectional bacterial envelope** spanning vertically with labeled compartments on left:

1. Outside the cell
2. Outer membrane
3. Periplasmic space
4. Inner membrane / Transmembrane
5. Cytoplasm

Use Gram-negative architecture:

- outer membrane with LPS
- periplasm
- inner membrane
- cytoplasm

Mechanism 1: Biofilm

Place at top.

Show tangled extracellular matrix fibers above membrane.

Label:
1 Biofilm

Visual:
Gray EPS mesh covering bacterial surface.

Mechanism 2: LPS Modification

Show LPS chains on outer membrane.

Label:
2 LPS modification

Illustrate:

- modified lipid A / LPS
- antibiotic approaching but blocked
- arrow to red X

Meaning:
Reduced binding of polymyxins / membrane-targeting drugs.

Mechanism 3: Porin Open

Show functional porin channel.

Label:
3 Porin (open)

Show:
Antibiotic molecule entering through channel.

Arrow downward into periplasm.

Mechanism 4: Porin Loss

Show closed/mutated porin.

Label:
4 Porin loss

Show:
Channel gray and blocked with red X.

Outcome:
Reduced drug influx.

Mechanism 5: Enzymatic Drug Inactivation

Large central orange-bordered box.

Header:
Enzymatic Drug Inactivation

Show three rows.

Hydrolysis

Label:
Hydrolysis – β-lactamases / Carbapenemases

Visual:
β-lactam ring → enzyme scissors → hydrolyzed inactive product.

Acetylation

Label:
Acetylation – AAC enzymes

Visual:
Drug + acetyl group (CH_3CO) → modified drug.

Phosphorylation / Adenylylation

Label:
Phosphorylation / Adenylylation – APH / ANT enzymes

Visual:
Drug + PO_4 or AMP attached.

Bottom note:
Modified drug loses target affinity

Mechanism 6–8: Efflux Pumps

Bottom membrane section.

5 RND efflux

Label:
5 RND efflux (AcrAB-TolC / MexAB-OprM / AdeABC)

Show tripartite pump spanning membranes.

Arrow outward.

---

6 ABC efflux

Label:
6 ABC efflux (MacB / MexXY / KpnGH)

Show ATP → ADP.

---

7 MFS efflux

Label:
7 MFS efflux (EmrAB / Tet systems)

Show proton-coupled export.

8 MATE/SMR efflux

Label:
8 MATE/SMR efflux (KmrA / KpnEF)

Show Na^+ coupled export.

Mechanism 9: gyrA / parC Mutation

Bottom cytoplasm.

Label:
9 gyrA / parC mutations

Visual:
DNA gyrase icon with antibiotic unable to bind.

Red X.

Mechanism 10: Ribosomal Methylation

Label:
10 Ribosomal methylation

Show ribosome with CH_3 group attached.

Antibiotic blocked.

Mechanism 11: Altered PBP2a

Label:
11 Altered PBP2a (mecA)

Show altered penicillin-binding protein.

β-lactam blocked.

Mechanism 12: PG Remodeling

Label:
12 PG remodeling (vanA/vanB)

Show peptidoglycan bricks.

Arrow toward modified target.

Red star for resistance.

Mechanism 13: Horizontal Gene Transfer

Label:
13 HGT / Plasmid

Show:
Plasmid circle → chromosome.

Arrow indicating gene acquisition.

PANEL B — FUNGAL CELL

Header:
B | FUNGAL CELL

Layout

Show fungal cell cross-section with:

- cell wall
- plasma membrane (ergosterol)
- cytoplasm

Left side labels:

- Outside / Cell wall
- Plasma membrane
- Cytoplasm

Mechanism 1: Biofilm

Top.

Label:
1 Biofilm

Gray extracellular fungal matrix.

Mechanism 2: FKS1 Mutation

Label:
2 FKS1 mutation

Show glucan synthase target.

Echinocandin star blocked.

Red X.

Mechanism 3: CDR1/2 Efflux

Label:
3 CDR1/2 ABC efflux

Show ABC transporter exporting azole.

Arrow upward.

ATP → ADP.

Mechanism 4: MDR1 Efflux

Label:
4 MDR1 MFS efflux

Show MFS pump.

H^+ dependent export.

Mechanism 5: Reduced Influx

Label:
5 Reduced influx

Show fewer drug molecules entering.

Arrow downward.

Mechanism 6: ERG11 Mutation

Label:
6 ERG11 mutation

Show azole target altered.

Red X.

Mechanism 7: ERG3 Loss

Label:
7 ERG3 loss

Show altered sterol pathway.

Outcome:
Sterol bypass.

Mechanism 8: Stress Response Pathway

Bottom central.

Label:
8 Stress-response pathway

Show signaling cascade:

Hsp90 → Calcineurin → Mkc1/Cek1 → Drug tolerance

Arrows between each.

Mechanism 9: TF Upregulation

Bottom large green oval.

Label:
9 TF upregulation

Inside box:
Tac1 / Mrr1 / Upc2 / Pdr1

Arrow to:
CDR1 / ERG11

Mechanism 10: Aneuploidy

Label:
10 Aneuploidy → ERG11 overexpression

Show duplicated chromosomes.

One chromosome highlighted red.

Mechanism 11: cyp51A Mutation

Label:
11 cyp51A mutation (TR34/L98H)

Show DNA mutation lightning symbol.

Mechanism 12: 5-FC Resistance

Label:
12 5-FC resistance mutations (FCY1/FCY2/FUR1)

Show 5-FC drug failing to act.

Red X.

PANEL C — VIRAL RESISTANCE

Header:
C | VIRAL RESISTANCE

Divide panel horizontally into:

Upper half:

ZONE A — RNA VIRUSES
(red border)

Lower half:

ZONE B — DNA VIRUSES
(purple border)

ZONE A — RNA VIRUSES

Mechanism 1

Label:
1 RdRp — error-prone replication

Show polymerase copying RNA with many mutations.

Arrow → diverse variants.

Mechanism 2

Label:
2 RdRp mutation — nucleoside analog resistance

Show nucleoside analog drug blocked.

Red X.

Mechanism 3

Label:
3 3CL protease mutation

Show protease mutation.

Protease inhibitor blocked.

Mechanism 4

Label:
4 RT — HIV lacks proofreading

Show reverse transcriptase making error-prone DNA.

Arrow to mutated DNA.

Mechanism 5

Label:
5 Selection → resistant variant

Show:
Sensitive virus (red X)
Resistant virus (green check)

Arrow:
Selection pressure.

ZONE B — DNA VIRUSES

Mechanism 6

Label:
6 TK mutation → prodrug failure

Show:
Prodrug (acyclovir) → mutated TK → no activation

Red X.

Mechanism 7

Label:
7 DNA pol mutation

Show polymerase mutation.

Drug cannot bind.

Mechanism 8

Label:
8 HCMV resistance (UL97 / UL54)

Show:

- ganciclovir blocked
- foscarnet blocked

Red X.

Mechanism 9

Label:
9 Cross-resistance

Show:
Acyclovir ↔ Foscarnet

Lightning symbol in center.

Mechanism 10

Label:
10 DNA pol proofreading → slower resistance

Show DNA polymerase with proofreading.

Arrow to slow evolution.

Bottom Legend (Full Width)

Create a legend row across bottom.

Header:
LEGEND (Shared Symbols)

Include icons with labels:

- Scissors = Enzymatic inactivation
- Lightning = Target site alteration
- Curved arrow = Efflux pump
- Gray oval + red X = Reduced permeability
- Gray fibers = Biofilm
- Chromosome = Genetic / chromosomal change
- Solid arrow = Active process
- Dashed arrow + red X = Inhibited / blocked process
- Blue hexagon = β-lactam
- Purple pentagon = Aminoglycoside / inhibitor
- Green triangle = Azole
- Red star = Echinocandin / vancomycin
- Orange diamond = Fluoroquinolone
- Blue capsule = Nucleoside analog
- Cyan diamond = Prodrug (Acyclovir)

Figure 2. Nanomedicine-mediated mechanisms for overcoming antimicrobial resistance. prompt in NotebookLM

Prompt:

Create a publication-quality scientific schematic figure illustrating the major mechanisms by which nanomedicine / antimicrobial nanoparticles (NPs) overcome antimicrobial resistance.

GENERAL FIGURE SETTINGS

Dimensions: 140 mm (width) × 90 mm (height)

Orientation: Horizontal landscape

Layout: Central hub-and-spoke design with 8 surrounding mechanism panels

Resolution: Minimum 300 dpi (journal-ready)

Font: Arial; all labels readable at final journal size

Style: Clean scientific infographic with high-quality vector icons

Background: White or soft light-gray with minimal clutter

Important: Do NOT include any figure title/header inside the image (journal does not allow embedded figure titles)

Color Palette

Central nanomedicine hub: metallic blue–silver gradient

Mechanism panels: use soft contrasting pastel colors for easy differentiation

Arrows: dark blue, bold, uniform thickness

Central Hub

Place a large central spherical nanoparticle hub labeled:

Nanomedicine / Antimicrobial NPs

Around the hub, display small representative NP examples arranged radially:

Liposome

Polymeric nanoparticle

Metallic nanoparticle (Ag/Au)

Dendrimer

Nanomicelle

Solid lipid nanoparticle

These serve as examples of nanomedicine platforms, not mechanism labels.

From the central hub, draw 8 bold outward arrows symmetrically toward each mechanism panel.

Mechanism Panels

1. Enhanced Membrane Penetration (Top Left)

Create a detailed membrane-transport schematic.

Illustrate:

Thick bacterial phospholipid bilayer with membrane proteins

Free antibiotic outside membrane showing blocked/poor penetration

Drug-loaded nanoparticle approaching membrane

NP fusing or penetrating membrane

Drug released into cytoplasm after entry

Add visual emphasis:

Red cross on poorly penetrating free drug

Green arrows showing successful NP-mediated transport

Label only:

Enhanced Membrane Penetration

Visual focus:

Clearly show nanoparticles bypassing permeability barriers.

2. Biofilm Disruption (Top Center Left)

Create a layered biofilm structure.

Illustrate:

Thick extracellular polymeric substance (EPS) mesh

Bacteria trapped inside dense matrix

Free drug unable to penetrate outer biofilm layers

Nanoparticles penetrating deeply through channels

Matrix breaking apart into fragments

Add details:

Broken EPS fibers

Detached bacteria

Penetration arrows reaching inner biofilm

Label only:

Biofilm Disruption

Visual focus:

Clearly show deep NP penetration and matrix collapse.

3. Efflux Pump Inhibition (Top Center Right)

Create a membrane protein inhibition schematic.

Illustrate:

Bacterial membrane with large efflux pump spanning bilayer

Free antibiotic being actively expelled outward

Nanoparticle docking at pump entrance

Pump channel blocked by NP or inhibitor cargo

Add details:

Outward red arrows for drug extrusion

Red inhibition symbol over pump

Drug retained inside cell after pump inhibition

Label only:

Efflux Pump Inhibition

Visual focus:

Clearly show drug retention caused by pump blockade.

4. ROS Generation (Top Right)

Create oxidative stress damage schematic.

Illustrate:

Reactive nanoparticle near microbial cell

Generation of ROS species:

•OH

O_2^-

H_2O_2

ROS attacking:

membrane

proteins

DNA

Add damage visuals:

Membrane rupture

DNA fragmentation

Protein oxidation

Lightning/oxidative burst symbols

Label only:

ROS Generation

Visual focus:

Clearly show multi-target oxidative destruction.

5. Drug Protection from Enzymatic Degradation (Bottom Right)

Create before-vs-after comparison.

Left side:

Free antibiotic exposed to β-lactamase

Enzyme degrading drug

Right side:

Drug encapsulated inside nanoparticle shell

Enzyme unable to access drug cargo

Add details:

Enzyme drawn like molecular scissors

Broken drug fragments on left

Intact encapsulated cargo on right

Label only:

Drug Protection

Visual focus:

Clearly show encapsulation shielding antibiotics from enzymes.

6. Stimuli-Responsive Release (Bottom Center Right)

Create infection-triggered smart release schematic.

Illustrate:

Intact nanocarrier circulating in normal conditions

Infection microenvironment containing:

acidic pH

ROS/redox trigger

enzymes

Trigger causes nanocarrier shell opening

Drug released only at infection site

Add details:

pH icon

Trigger switch symbol

Burst-release arrows

Label only:

Stimuli-Responsive Release

Visual focus:

Clearly show site-specific triggered release.

7. Intracellular Targeting (Bottom Center Left)

Create infected host-cell targeting schematic.

Illustrate:

Mammalian host cell membrane

Intracellular pathogen inside endosome/cytoplasm

Free drug remains extracellular

Nanoparticle enters via endocytosis

NP releases drug close to intracellular pathogen

Add details:

Endocytosis vesicle

Intracellular bacteria or virus

Release arrows toward pathogen

Label only:

Intracellular Targeting

Visual focus:

Clearly show delivery to hidden intracellular pathogens.

8. Combination Therapy / Co-delivery (Bottom Left)

Create synergistic delivery schematic.

Illustrate:

Single nanoparticle with dual compartments

Cargo 1: antibiotic molecule

Cargo 2: adjuvant / metallic NP / efflux inhibitor

After release:

Both cargos attack different targets simultaneously:

membrane

DNA

efflux pump

metabolism

Add details:

Two distinct cargo colors

Multiple attack arrows

Synergy symbol

Label only:

Combination Therapy

Visual focus:

Clearly show simultaneous multi-target attack.

Final Figure Requirements

No figure title inside image

Use labels only (avoid descriptive sentences inside panels)

Mechanisms must be understandable primarily through visuals

Use large, detailed, professional icons

Maintain perfect radial symmetry

Central hub must remain visually dominant

Use clean spacing between panels

Output should resemble high-impact review figures

Figure 3. Schematic illustration of the multi-dimensional impact of antimicrobial nanomedicine on host biology, microbial resistance, microbiome stability, and environmental ecosystems.

Prompt in NotebookLM

Create a clean, publication-quality scientific figure in a square layout (140 × 140 mm) with balanced image and minimal text. Do not add any figure title above or summary text below. Avoid long explanatory text. Use icons, labels (1–3 words max), and directional arrows to represent pathways and cause–effect relationships.

Use a **central hub-and-spoke layout**.

Center

Place a central circle containing **Antimicrobial Nanoparticles (NPs)** showing representative nanoparticles:

- AgNP
- AuNP
- Metal oxide NP
- Liposome

- RNA nanoparticle

Around the center include only short labels:

- Small size
- High surface area
- High reactivity
- High penetration

Three major branches should emerge from the central NP hub.

Left Panel: Host Toxicity

Show human body silhouette with highlighted organs:

- Brain
- Lungs
- Liver
- Spleen
- Kidney
- Intestine

Show nanoparticle accumulation in organs.

Use arrow pathways only:

NPs → ROS ↑ → Oxidative stress → Lipid peroxidation
NPs → Mitochondrial damage
NPs → DNA damage
NPs → Inflammation
NPs → Apoptosis
NPs → Behavioral changes

Use icons for ROS, damaged mitochondria, broken DNA, inflammatory cells, apoptotic cells.

Upper Right Panel: Microbiome Disruption + Resistance

Show healthy gut microbiota transitioning to dysbiosis.

Arrow pathway:

Healthy microbiota → NP exposure → Dysbiosis

Below this show:

Dysbiosis → Immune dysregulation
Dysbiosis → Reduced nutrient absorption
Dysbiosis → Altered metabolism

Next to gut, show bacterial adaptation.

Arrow pathways:

NP exposure → Genetic mutation
NP exposure → Metal efflux pumps ↑
NP exposure → Reduced uptake
NP exposure → Membrane remodeling
NP exposure → Biofilm formation
NP exposure → ROS adaptation

Show plasmid icon.

Arrow:
Metal tolerance genes ↔ Antibiotic resistance genes → Co-selection of AMR

Bottom Panel: Environmental Fate

Show pathway using icons and arrows only:

Production → Clinical use → Disposal → Wastewater → Soil / Water

Then:

NPs → Transport
→ Aggregation
→ Dissolution
→ Sedimentation
→ Bioaccumulation

Show ecosystem transfer using arrows:

Microbes → Earthworm → Plant → Fish → Bird/Animal

Add ecological consequences via arrows:

NP exposure → Microbial shift
NP exposure → Nutrient cycling disruption
NP exposure → Oxidative stress
NP exposure → Poor seed germination
NP exposure → Reduced plant growth

Final arrow:

Environmental persistence → NP-resistant strains

Design Requirements

- No title above figure
- No summary text below figure
- If showing process, use arrows instead of descriptive sentences
- Maximum label length: 1–3 words

- Minimal clutter
- High readability at 140 mm
- Use soft scientific colors:
    - Red/orange = toxicity
    - Green = healthy/ecology
    - Blue/gray = nanoparticles/environment
- Use vector scientific icons only
- Final figure should resemble a high-impact review journal graphical illustration
